# Preclinical Evaluation of Combined Polo-like Kinase 1 Inhibition and Navitoclax in Experimental Models of Lung Cancer

**DOI:** 10.3390/ijms27167359

**Published:** 2026-08-17

**Authors:** Bárbara Pinto, Mateus Prates-Rodrigues, Daniel Augusto Barnabe Nobre, Mariana de Oliveira Silva, Bárbara M. de Amorim-Santos, João P. N. Silva, Patrícia M. A. Silva, Bruno Sarmento, Marisa Salvi, Geovanni Dantas Cassali, Elaine Maria de Souza-Fagundes, Hassan Bousbaa, Juliana Carvalho-Tavares

**Affiliations:** 1 UNIPRO—Oral Pathology and Rehabilitation Research Unit, University Institute of Health Sciences (IUCS), Cooperativa de Ensino Superior Politécnico e Universitário (CESPU), 4585-116 Gandra, Portugal; joaosilva_06@hotmail.com (J.P.N.S.); patricia.silva@cespu.pt (P.M.A.S.); hassan.bousbaa@iucs.cespu.pt (H.B.); 2 Department of Physiology and Biophysics, Institute of Biological Sciences, Federal University of Minas Gerais (UFMG), Av. Pres. Antônio Carlos, 6627, Belo Horizonte 31270-901, Brazil; mateus.prates120@gmail.com (M.P.-R.); daniel_nobre@yahoo.com (D.A.B.N.); marrioliveira15@gmail.com (M.d.O.S.); elainefagundes@icb.ufmg.br (E.M.d.S.-F.); 3 UCIBIO—Applied Molecular Biosciences Unit, Translational Toxicology Research Laboratory, University Institute of Health Sciences (1H-TOXRUN, IUCS-CESPU), 4585-116 Gandra, Portugal; 4 Department of Microbiology, Institute of Biological Sciences, Federal University of Minas Gerais (UFMG), Av. Pres. Antônio Carlos, 6627, Belo Horizonte 31270-901, Brazil; bmamorims@gmail.com; 5 Associate Laboratory i4HB—Institute for Health and Bioeconomy, University Institute of Health Sciences—CESPU, 4585-116 Gandra, Portugal; 6 i3S-Institute for Research and Innovation in Health, University of Porto, Rua Alfredo Allen 208, 4200-135 Porto, Portugal; bruno.sarmento@iics.cespu.pt; 7 INEB-Institute of Biomedical Engineering, University of Porto, Rua Alfredo Allen 208, 4200-393 Porto, Portugal; 8 Department of General Pathology, Institute of Biological Sciences, Federal University of Minas Gerais (UFMG), Av. Pres. Antônio Carlos, 6627, Belo Horizonte 31270-901, Brazil; salvimarisa21@gmail.com (M.S.); geovanni.cassali@gmail.com (G.D.C.)

**Keywords:** PLK-1, BI2536, Navitoclax, combination therapy, lung cancer animal models

## Abstract

Targeted therapies have substantially advanced cancer treatment; however, therapeutic resistance remains a major challenge, particularly in lung cancer. Polo-like kinase 1 (PLK-1), a key regulator of mitosis, is frequently overexpressed in lung tumors and has emerged as an attractive anticancer target. Although PLK-1 inhibition induces robust antitumor effects in preclinical models, its translation into clinical benefit has been limited, particularly when used as monotherapy. Previous in vitro work from our group demonstrated that combining PLK-1 inhibition with the pro-apoptotic agent Navitoclax markedly enhanced cancer cell death by reducing mitotic slippage and promoting post-mitotic apoptosis. Here, we investigated whether this combinatorial strategy translates into therapeutic benefit in vivo. Using Lewis lung carcinoma (LLC1) cells, we evaluated the effects of BI2536 (a PLK-1 inhibitor) and Navitoclax, alone or in combination, in vitro and in male C57BL/6J mice using three lung cancer models: subcutaneous, intranasal, and intrapulmonary. The in vitro results corroborated the findings of our previous work, demonstrating synergistic effects. In the murine models, tumor progression, body weight, survival, and histopathological features were assessed to evaluate therapeutic efficacy and systemic toxicity. In the subcutaneous model, BI2536 monotherapy significantly reduced tumor volume compared with vehicle-treated controls, while Navitoclax alone and the combination therapy also produced tumor growth inhibition. In contrast, in the intranasal model, no significant differences were observed in body weight or survival among groups. Nonetheless, treatment with BI2536 alone or in combination with Navitoclax significantly reduced tumor cell proliferative activity. In the aggressive intrapulmonary (orthotopic) model, rapid disease progression and high mortality were observed across all groups. Notably, BI2536 treatment was associated with improved survival compared with Navitoclax monotherapy, whereas the combination therapy did not confer additional survival benefit. Histological analyses revealed highly proliferative tumors with pronounced cellular and nuclear atypia. Moreover, the BI2536 monotherapy group exhibited significantly greater necrosis/apoptosis than the other treatment groups, while diffuse and focal hepatic steatosis was observed exclusively in the combination group. Together, these findings indicate that while PLK-1 inhibition exerts antitumor effects in certain in vivo contexts, the therapeutic synergy observed in vitro with Navitoclax does not consistently translate to in vivo lung cancer models. This study highlights the challenges of translating combinatorial mitotic and apoptotic targeting strategies into effective in vivo therapies and underscores the importance of model selection and tumor microenvironment in preclinical drug evaluation.

## 1. Introduction

Lung cancer remains the leading cause of cancer-related mortality worldwide, accounting for approximately 1.8 million deaths annually and representing a major global health burden. Among lung cancer types, non-small cell lung cancer (NSCLC) is the most prevalent form, comprising approximately 85% of all cases and continuing to exhibit poor overall survival rates despite advances in early detection and therapy [1,2,3].

In recent years, advancements in treatment strategies for NSCLC, particularly targeted therapies and immunotherapy, have improved clinical outcomes in selected patient populations. Targeted therapies directed against molecular alterations such as EGFR, ALK, BRAF, MET, and other oncogenic drivers have demonstrated clinical benefits [4]. However, the overall five-year survival rate for NSCLC patients remains low, ranging from approximately 15% to 20% [5,6]. These limitations of current treatment modalities highlight the urgent need for the development of novel therapeutic strategies to improve long-term outcomes in NSCLC.

Dysregulation of cell cycle control is a hallmark of cancer, and mitotic kinases have emerged as attractive therapeutic targets. Among them, Polo-like kinase 1 (PLK-1) is a serine/threonine kinase that plays a critical role in multiple stages of mitosis, including centrosome maturation, spindle assembly, chromosome segregation, and cytokinesis [7,8,9,10,11]. PLK-1 expression is minimal in most differentiated normal tissues but significantly upregulated in many malignancies, including lung cancer, where its elevated expression correlates with aggressive tumor behavior and poor patient prognosis [7,12,13,14,15]. Preclinical studies have demonstrated that PLK-1 inhibition disrupts mitotic progression, leading to mitotic arrest and cell death in lung cancer cells [16,17]. However, despite encouraging results in vitro, PLK-1 inhibitors used as single agents have shown modest clinical efficacy, possibly due to mitotic slippage, adaptive survival mechanisms, and dose-limiting toxicities [18,19,20].

To overcome these limitations, increasing attention has been directed toward combination therapies that exploit mitotic vulnerabilities while simultaneously blocking pro-survival pathways. One promising strategy involves combining PLK-1 inhibition with agents that target intrinsic apoptotic machinery. Navitoclax (ABT-263) is a small-molecule inhibitor of anti-apoptotic BCL-2 family proteins, including BCL-2, BCL-XL, and BCL-W, and has demonstrated potent pro-apoptotic activity in various cancer models [21]. Nevertheless, Navitoclax monotherapy has shown limited efficacy in solid tumors, including lung cancer, due to intrinsic resistance mechanisms and on-target toxicities such as thrombocytopenia [22]. In a previous study from our group, we demonstrated that the combined inhibition of PLK-1 (BI2536) and BCL-2 family proteins using Navitoclax synergistically induces pronounced cell death in lung cancer cells. This combination promoted post-mitotic cell death, significantly reduced mitotic slippage, and enhanced apoptotic signaling compared to either agent alone, highlighting a strong mechanistic rationale for this therapeutic approach [23].

Here, we extend these findings by evaluating the therapeutic efficacy and systemic tolerability of BI2536 and Navitoclax, administered alone or in combination, in complementary murine models of lung cancer. Lewis lung carcinoma (LLC1) cells were inoculated using subcutaneous, intranasal and intrapulmonary approaches, enabling the evaluation of treatment responses across distinct patterns of tumor growth and pulmonary disease progression. The subcutaneous model enabled accurate longitudinal assessment of tumor growth, whereas the intranasal and intrapulmonary models more closely recapitulate the pulmonary tumor microenvironment and disease progression. Together, these complementary models provide a comprehensive preclinical framework for evaluating the antitumor efficacy of PLK1 inhibition combined with apoptotic priming.

## 2. Results

### 2.1. Navitoclax Potentiates the Cytotoxic Activity of BI2536 and Selectively Sensitizes Murine Lung Cancer Cells

**Figure 1:**
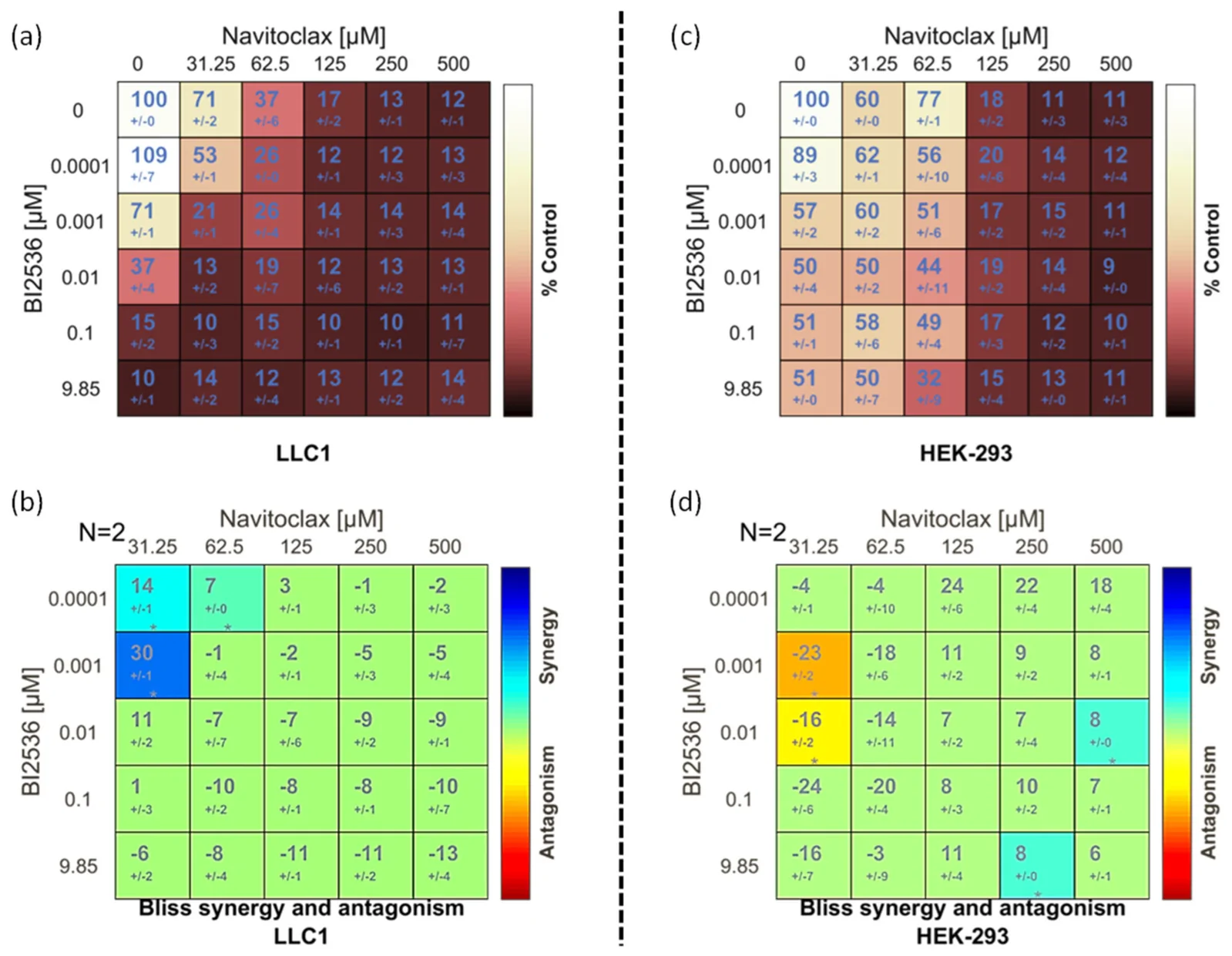
Navitoclax enhances the cytotoxic activity of BI2536 in LLC1 murine lung cancer cells while exhibiting reduced activity in HEK-293 cells. LLC1 and HEK-293 cells were treated with increasing concentrations of navitoclax and BI2536, either as single agents or in combination, for 48 h. (**a**,**b**) Cell viability (%) following treatment with the indicated drug concentrations, determined by the MTT assay from two independent biological experiments, each performed in technical triplicate. (**c**,**d**) Drug interaction maps generated using Combenefit software (version 2.021) based on the Bliss independence model, showing Bliss synergy scores across the tested concentration matrix. Positive Bliss scores indicate synergistic interactions, values close to zero indicate additive effects, and negative scores indicate antagonistic interactions. Synergistic (blue), additive (green), and antagonistic (red) interactions are represented according to the calculated Bliss scores. Asterisks indicate statistically significant synergistic or antagonistic interactions. Statistical significance of the synergy scores is indicated as * *p* < 0.05. IC_50_ values were calculated by nonlinear regression using the log(inhibitor) vs. normalized response (variable slope) model in GraphPad Prism 8.0 and are presented as mean ± SD.

Based on our previous study demonstrating that navitoclax potentiates the antitumor activity of the PLK1 inhibitor BI2536 in A549 and NCI-H460 human lung cancer cells, we next investigated whether this therapeutic interaction could also be reproduced in the murine lung cancer cell line LLC1. LLC1 cells were treated with increasing concentrations of navitoclax and BI2536, either alone or in combination, for 48 h, and cell viability was assessed using the MTT assay. Drug interactions were evaluated using Combenefit software. Consistent with our previous findings, the combination treatment reduced LLC1 cell viability more effectively than either drug alone. Combenefit analysis revealed synergistic interactions at specific concentration pairs, whereas most of the remaining combinations displayed additive effects (Figure 1a,c). No antagonistic interactions were observed. The IC_50_ values for navitoclax and BI2536 were 43.21 ± 4.59 μM and 0.0025 ± 0.0005 μM, respectively (Table 1).

The same experimental approach was performed in HEK cells, a non-carcinogenic and embryonic complementary human-derived cell model to provide an additional assessment of general cytotoxicity. Most drug combinations resulted in additive effects, although a limited number of synergistic interactions were also identified. In contrast to LLC1 cells, antagonistic interaction points were detected in HEK cells (Figure 1b,d). Furthermore, HEK cells were less sensitive to both drugs, exhibiting higher IC_50_ values for navitoclax (74.44 ± 1.75 μM) and BI2536 (>9.85 µM) than LLC1 cells (Table 2). These findings suggest that LLC1 cells are more sensitive to both single-agent and combination treatments than HEK cells.

### 2.2. Standardization and Characterization of Murine Models of Lung Cancer

To establish and standardize murine models of lung cancer, LLC1 cells were inoculated using subcutaneous, intranasal, and intrapulmonary routes. In all models, animals received 1.0 × 10^6^ LLC1 cells on day 0 and were subsequently monitored for body weight variation, clinical parameters, tumor progression, and survival throughout the experimental period.

**Figure 2:**
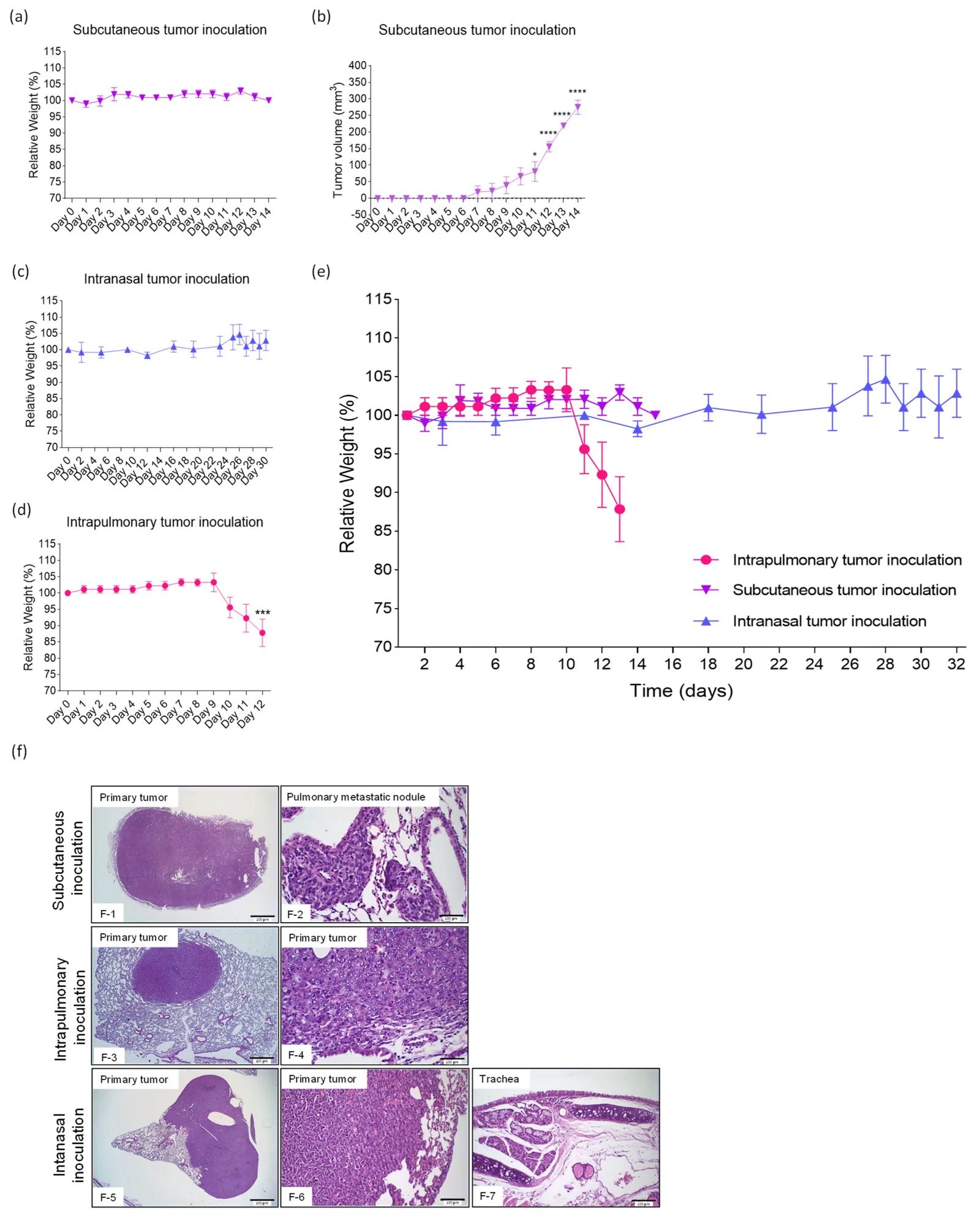
Establishment and standardization of murine lung cancer models using different inoculation routes. (**a**) Relative body weight of animals subjected to subcutaneous LLC1 tumor inoculation monitored throughout the experimental period. (**b**) Tumor volume progression in the subcutaneous model measured by digital calipers over time. (**c**) Relative body weight of animals following intranasal LLC1 cell inoculation monitored during the experimental period. (**d**) Relative body weight of animals subjected to intrapulmonary LLC1 tumor inoculation monitored over time. (**e**) Comparative representation of relative body weight variation across subcutaneous, intranasal, and intrapulmonary lung cancer models. (**f**) Representative hematoxylin and eosin-stained histological sections of lung tissues obtained from subcutaneous, intrapulmonary and intranasal models, as well as tracheal sections from the intranasal model. Subcutaneous inoculation: F-1 Primary tumor (2×) and F-2 Pulmonary metastasis (40×); Intrapulmonary inoculation: F-3 Primary tumor (2×) and F-4 (40×); Intranasal inoculation: Primary tumor; F-5 (2×) and F-6 (40×) F-7 Trachea (20×). Data are presented as mean ± SEM; *n* = 5 animals per group. Statistical analyses were performed using mixed-effects models for body weight and tumor volume, and Log-rank (Mantel–Cox) test for survival. Significance is indicated as * *p* < 0.05, *** *p* < 0.001, **** *p* < 0.0001.

In the subcutaneous model, body weight remained stable over time, with no significant variations observed between experimental days (Figure 2a). Tumor progression was evident and quantifiable in this model, as tumor volume increased progressively following inoculation (Figure 2b). A significant increase in tumor size was detected from day 11 (*p* < 0.05) onward compared with day 0, reaching approximately 100 mm^3^ and continuing to expand until the experimental endpoint on day 14, confirming consistent tumor establishment.

In the intranasal model, no significant alterations in body weight were observed during the experimental period (Figure 2c).

In the intrapulmonary model, a progressive reduction in relative body weight was observed over time (Figure 2d). Notably, after day 9 post-inoculation, animals exhibited a more pronounced weight loss, which became statistically significant by day 12 when compared to baseline values (day 0), with a highly significant difference (*p* < 0.001). This weight loss was accompanied by worsening clinical signs, including apathy, reduced locomotor activity, and decreased interaction with the environment, indicating disease progression and the systemic impact of intrapulmonary tumor growth.

A comparative analysis integrating body weight data from all three inoculation routes is presented in Figure 2e. Clear differences in disease progression were observed, with the intrapulmonary model showing the earliest and most severe systemic effects, whereas the subcutaneous and intranasal models displayed more localized tumor development.

Regarding the histopathological evaluation using H&E staining, tumor presence was found in all experimental models (Figure 2f), demonstrating successful tumor induction protocols. In the intranasal model, tracheal sections remained free of tumor cells, reinforcing that cancer cells were effectively delivered to the lungs without upper airway involvement. Together, these findings validate the robustness and translational relevance of the three lung cancer models for subsequent therapeutic efficacy and toxicity studies.

### 2.3. Therapeutic Effects of PLK-1 Inhibition and Navitoclax in a Subcutaneous Lung Cancer Model

**Figure 3:**
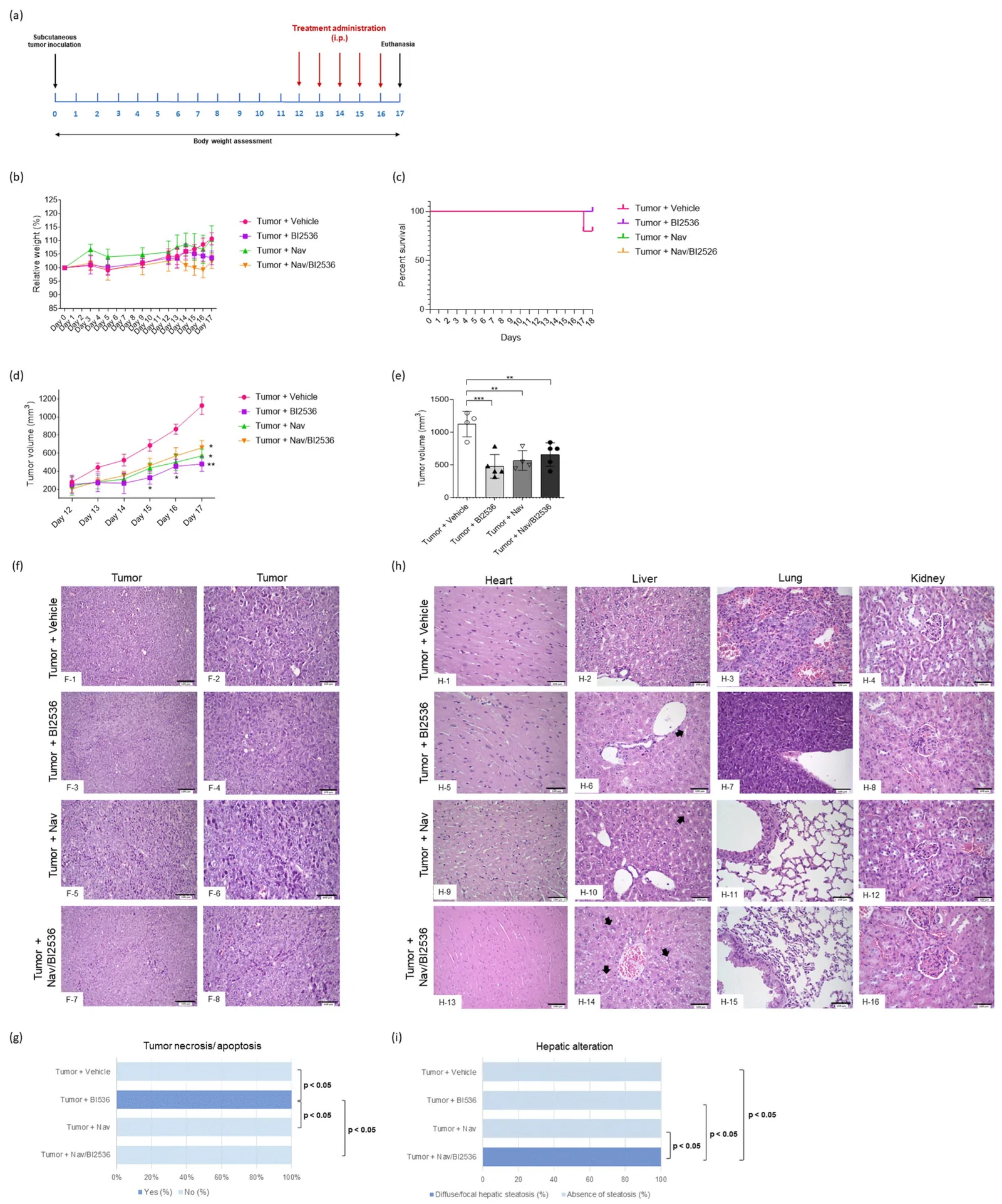
Therapeutic evaluation of Navitoclax and BI2536 in the subcutaneous LLC1 lung cancer model. (**a**) Experimental timeline showing tumor inoculation, treatment schedule (day 12–16), and endpoint (day 17). (**b**) Relative body weight of tumor-bearing animals throughout the treatment period. (**c**) Kaplan–Meier survival curves of animals in each experimental group. (**d**) Longitudinal tumor volume measurements during the treatment period. (**e**) Tumor volumes at day 17 (endpoint). (**f**) Representative hematoxylin and eosin-stained tumor sections. F-1, F-3, F-5 and F-7 Primary tumor (20×); F-2, F-4, F-6 and F-8 Primary tumor (40×). (**g**) Quantification of necrosis and apoptotic figures in the tumor + BI2536 group. (**h**) Representative 40× photomicrographs of heart, spleen, lung, and kidney. (**i**) Liver histopathology showing diffuse and focal steatosis in the combination-treated group. Data are presented as mean ± SEM; *n* = 4 (Tumor + Vehicle), 5 (Tumor + Navitoclax), 5 (Tumor + BI2536), 5 (Tumor + Navitoclax + BI2536). Statistical analyses were performed using mixed-effects models for repeated measurements, one-way ANOVA followed by Tukey’s post hoc test for tumor volume at endpoint, and Log-rank (Mantel–Cox) test for survival. Differences in the frequency of apoptosis/necrosis and of hepatic steatosis among experimental groups were analyzed using Fisher’s exact test with Monte Carlo simulation, followed by pairwise Fisher tests with false discovery rate correction. Significance is indicated as * *p* < 0.05, ** *p* < 0.01, *** *p* < 0.001.

Following the establishment of the subcutaneous LLC1 lung cancer model, therapeutic interventions were initiated based on tumor progression kinetics defined during model characterization. As illustrated in Figure 2b,e, subcutaneous tumors reached an average volume of approximately 150–200 mm^3^ on day 12 post-inoculation, which was selected as the starting point for treatment. Animals then received five consecutive days of treatment, from day 12 to day 16, according to the experimental design shown in Figure 3a, and were euthanized on day 17.

To assess systemic tolerability, body weight variation was monitored throughout the treatment period. Our data evidenced that relative body weight remained stable over time in all experimental groups (Figure 3b). No statistically significant differences in body weight were observed among groups during the treatment period, suggesting that neither monotherapy nor combination therapy induced overt systemic toxicity, as assessed by body weight, under the experimental conditions. Animals were monitored throughout the treatment period until the predefined experimental endpoint (day 17). As shown in Figure 3c, one unscheduled death occurred in the Tumor + Vehicle group on day 17, whereas the remaining animals reached the predefined experimental endpoint.

Tumor progression was assessed by longitudinal measurement of tumor volume. Tumor-bearing animals treated with BI2536 alone exhibited a significant reduction in tumor volume compared to vehicle-treated controls on day 17 (*p* < 0.05) (Figure 3d). In addition, a statistically significant difference (*p* < 0.05) was also observed between tumor + BI2536 group and tumor + vehicle group on days 16 and 17, indicating a sustained inhibitory effect of PLK-1 inhibition on tumor growth during the late treatment phase. Treatment with Navitoclax alone or in combination therapy with BI2536 also resulted in a statistically significant reduction in tumor volume compared to the vehicle-treated group (*p* < 0.05) (Figure 3d). To further compare treatment effects at the experimental endpoint, tumor volumes from all groups were analyzed specifically on day 17 (Figure 3e). The results revealed a highly significant reduction in tumor volume in the tumor + BI2536 group compared to the tumor + vehicle group (*p* < 0.001). Additionally, the tumor + Navitoclax group and the tumor + Navitoclax + BI2536 combination group both exhibited significant reductions in tumor volume relative to the tumor + vehicle group (*p* < 0.01). No statistically significant differences were observed among the experimental treatment groups, indicating comparable antitumor efficacy at this endpoint.

Histopathological evaluation of subcutaneous tumors was performed to further characterize treatment-associated morphological changes. Representative hematoxylin and eosin-stained sections are shown in Figure 3f. All experimental groups exhibited tumors with a solid growth pattern, scant stromal component, and neoplastic cells displaying marked cellular and nuclear pleomorphism, along with frequent mitotic figures, including atypical mitoses. Notably, tumors from animals treated exclusively with BI2536 monotherapy also exhibited central areas of necrosis and occasional apoptotic figures, distinguishing this group from the others. These histopathological features were consistently observed in 100% of the animals analyzed, as quantitatively summarized in Figure 3g. Statistical analysis confirmed a significant difference in the occurrence of apoptosis/necrosis between the BI2536 monotherapy group and all other experimental groups (*p* < 0.05).

To assess potential off-target or systemic histopathological effects, additional organs were analyzed. Representative 40× photomicrographs of the heart, spleen, lung, and kidney from all experimental groups were performed (Figure 3h). No evidence of histological alterations was detected in the brain, heart, and kidneys after any of the treatment conditions. In the liver, no apparent morphological alterations were observed in the tumor + vehicle, tumor + BI2536, or tumor + Navitoclax groups. However, animals subjected to the combination therapy (Navitoclax + BI2536) exhibited both diffuse and focal hepatic steatosis, as quantitatively demonstrated (Figure 3i). Statistical analysis revealed that the frequency of hepatic steatosis was significantly higher in the combination therapy group compared with the other experimental groups (*p* < 0.05).

Together, these results demonstrate that PLK-1 inhibition using BI2536 exerts a measurable antitumor effect, inducing cellular apoptosis/necrosis and reducing tumor volume in the subcutaneous lung cancer model without inducing significant systemic toxicity. While Navitoclax alone or in combination with BI2536 effectively reduced tumor volume, the combination therapy did not provide additional antitumor benefit compared to monotherapies under the conditions evaluated and was associated with hepatic steatosis.

### 2.4. Therapeutic Effects of PLK-1 Inhibition and Navitoclax in the Intranasal Lung Cancer Model

**Figure 4:**
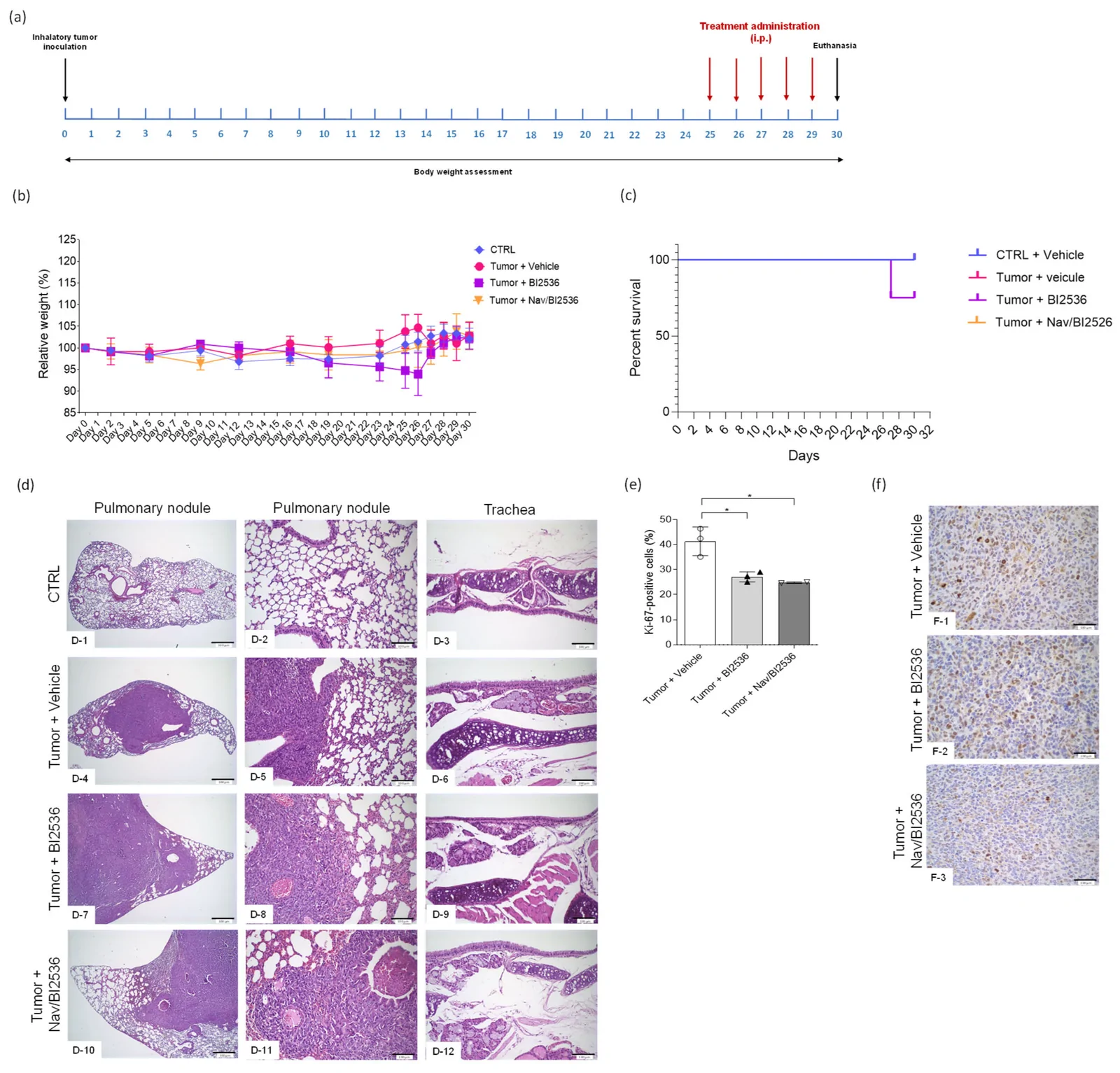
Therapeutic evaluation of PLK-1 inhibition and Navitoclax in the intranasal LLC1 lung cancer model. (**a**) Experimental timeline showing intranasal inoculation of LLC1 cells (day 0), treatment initiation (day 25), treatment period (day 25–29), and endpoint (day 30). (**b**) Relative body weight of animals monitored throughout the treatment period. (**c**) Kaplan–Meier survival curves of animals in each experimental group. (**d**) Representative hematoxylin and eosin–stained lung sections showing pulmonary architecture and tumor infiltration (D-1, D-4, D-7 and D-10 with 4× magnification; D-2; D-5, D-8 and D-11 with 20× magnification) as well as preserved tracheal structure without evidence of neoplastic cell accumulation (D-3, D-6, D-9 and D-12 with 20× magnification). Lung tissues from tumor-bearing groups exhibit extensive neoplastic areas with marked replacement of normal parenchyma and reduction in alveolar spaces. Data are presented as mean ± SEM; *n* = 5 (CTRL), 4 (Tumor + Vehicle), 4 (Tumor + BI2536), 4 (Tumor + Navitoclax + BI2536). (**e**) Percentage of Ki-67-positive cells. (**f**) Representative photomicrographs of Ki-67 immunostaining in tumors from the different experimental groups (F-1, F-2 and F-3). Ki-67 immunoreactivity is observed as brown nuclear staining in neoplastic cells, with hematoxylin counterstaining. Original magnification 40×. Statistical analyses were performed using mixed-effects models for repeated body weight measurements, the Log-rank (Mantel–Cox) test for survival analysis, and one-way ANOVA followed by Tukey’s post hoc test for Ki-67-positive cells evaluation. Significance is indicated as * *p* < 0.05.

To evaluate the therapeutic impact of PLK-1 inhibition and Navitoclax in a physiologically relevant inhalation model of lung cancer, LLC1 cells were administered intranasally on day 0. Treatment was initiated on day 26, once tumors were established, and continued for five consecutive days until day 31, as illustrated in Figure 4a. Animals were euthanized on day 32 for tissue collection and subsequent analyses.

Body weight variation was monitored throughout the treatment period as a measure of systemic tolerability. As shown in Figure 4b, no statistically significant differences in body weight were observed among the experimental groups. Survival was assessed during the treatment period and until the experimental endpoint. As shown in Figure 4c, one death occurred in the Tumor + Navitoclax/BI2536 group; however, overall survival rates did not differ significantly among the experimental groups, demonstrating that the treatments were generally well tolerated.

Histopathological evaluation of lung sections was performed to assess potential treatment-associated alterations (Figure 4d). No structural abnormalities were observed in the trachea across experimental groups, and no evidence of neoplastic cells was detected in tracheal tissue. These findings indicate that intranasal inoculation of LLC1 cells did not result in tumor formation or tumor cell accumulation in the trachea, supporting the effectiveness of this route in delivering cells directly to the pulmonary compartment. In the Tumor + Vehicle, Tumor + BI2536, and Tumor + Navitoclax/BI2536 groups, extensive areas of neoplastic tissue were observed, characterized by marked replacement of the normal lung parenchyma and a pronounced reduction in alveolar spaces. Across treated groups, no evident reduction in tumor mass was detected by histopathological assessment, indicating that therapeutic interventions did not substantially alter tumor burden at the tissue level in intranasal model.

Immunohistochemical evaluation of Ki-67 expression was performed to assess tumor cell proliferative activity (Figure 4e,f). As shown in Figure 4e, treatment with BI2536 alone or in combination with Navitoclax significantly reduced the percentage of Ki-67-positive cells compared with the Tumor + Vehicle group (*p* < 0.05). No significant differences were observed between the BI2536 monotherapy and combination therapy groups. Representative immunohistochemical staining is shown in Figure 4f.

Together, these results indicate that the intranasal LLC1 model reliably reproduces aggressive pulmonary tumor growth with extensive involvement of the lung parenchyma. Although treatment with BI2536 alone or in combination with Navitoclax did not significantly affect body weight, survival, or overall histopathological tumor burden under the conditions evaluated, BI2536-containing treatments significantly reduced the proportion of Ki-67-positive tumor cells, indicating decreased tumor cell proliferative activity.

### 2.5. Therapeutic Effects of PLK-1 Inhibition and Navitoclax in the Intrapulmonary Lung Cancer Model

**Figure 5:**
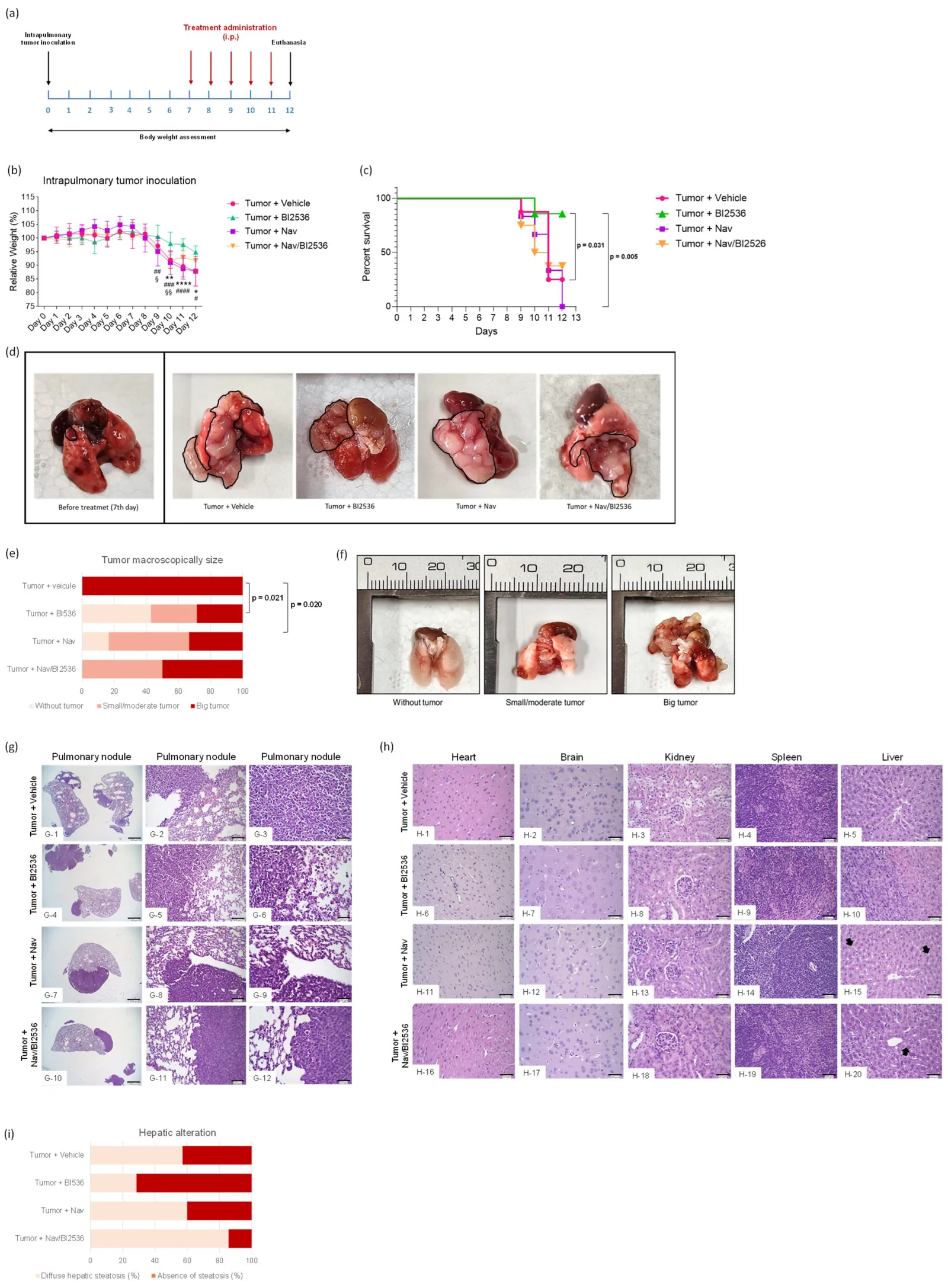
Therapeutic evaluation of PLK-1 inhibition and Navitoclax in the intrapulmonary LLC1 lung cancer model. (**a**) Experimental timeline showing intrapulmonary inoculation of LLC1 cells (day 0), treatment schedule, and endpoint (day 12 post-inoculation). (**b**) Relative body weight of animals monitored throughout the experimental period. (**c**) Kaplan–Meier survival curves of animals in each experimental group. (**d**) Representative images of lung tumors showing small tumor nodules at early stages, delineated by perimeter markings, followed by rapid expansion into large tumor masses at later time points. (**e**) Quantification of macroscopic tumor burden categorized as without tumor, small/moderate tumor, or large tumor. (**f**) Representative photographs of each macroscopic tumor category. (**g**) Histopathological evaluation of lung tissues showing aggressive tumor characteristics across all groups, including solid growth pattern, high cellularity, partial replacement of lung parenchyma, and compression of alveolar spaces. Neoplastic cells displayed marked cellular and nuclear pleomorphism, increased nucleocytoplasmic ratio, prominent nucleoli, and numerous mitotic figures. No apparent morphological differences were observed between treatment groups and the vehicle group (G-1, G-4, G-7 and G-10 with 2× magnification; G-2, G-5, G-8 and G-11 with 20× magnification; G-3, G-6, G-9 and G12 with 40× magnification). (**h**) Hepatic steatosis was observed in the Tumor + Navitoclax and Tumor + Navitoclax + BI2536 groups, as indicated by the arrows. No apparent alterations were observed in the other organs. H-1–H-20 with 40× magnification. (**i**) Hepatic evaluation showing the proportion of animals with diffuse hepatic steatosis across experimental groups. Data are presented as mean ± SEM; *n* = 8 (Tumor + Vehicle), 6 (Tumor + Navitoclax), 7 (Tumor + BI2536), 8 (Tumor + Navitoclax + BI2536). Statistical analyses were performed using mixed-effects models for repeated body weight measurements, the Log-rank (Mantel–Cox) test for survival analysis, and Fisher–Freeman–Halton test with pairwise comparisons for macroscopic tumor burden. Significance is indicated as * *p* < 0.05, ** *p* < 0.01, **** *p* < 0.0001 between Tumor + BI2536 and Tumor + Vehicle, $ *p* < 0.05, $$ *p* < 0.01 between Tumor + BI2536 and Tumor + Navitoclax + BI2536, and # *p* < 0.05, ## *p* < 0.01, ### *p* < 0.001, #### *p* < 0.0001 between Tumor + BI2536 and Tumor + Navitoclax.

To evaluate the therapeutic potential of PLK-1 inhibition and Navitoclax in a clinically relevant intrapulmonary lung cancer model, LLC1 cells were directly injected into the lung parenchyma on day 0. Treatment was administered according to the schedule depicted in Figure 5a, with interventions tailored to tumor establishment. Animals were monitored throughout the experimental period and euthanized on day 12 post-inoculation.

Significant differences in relative body weight were observed among treatment groups, particularly involving the Tumor + BI2536 group (Figure 5b). On day 9, animals treated with BI2536 exhibited an attenuation of body weight loss compared to the tumor + Navitoclax and combination therapy groups (*p* < 0.01 and *p* < 0.05, respectively). On day 10, the Tumor + BI2536 group differed significantly from the vehicle-treated tumor group and the combination therapy group (*p* < 0.01 for both comparisons), as well as from the Navitoclax-treated group (*p* < 0.001). On day 11, marked differences were observed between the Tumor + BI2536 group and both the vehicle-treated tumor group and the Navitoclax-treated group (*p* < 0.0001 for both comparisons). On day 12, a significant difference persisted between the Tumor + BI2536 group and the Tumor + vehicle and Tumor + Navitoclax groups (*p* < 0.05 for both comparisons). Given the differential mortality observed among the experimental groups, body weight comparisons at later time points represent the animals remaining alive at each respective assessment. During daily monitoring throughout the experimental period, animals treated with Navitoclax, either alone or in combination with BI2536, appeared more apathetic and displayed reduced spontaneous movement compared with the other groups. In contrast, mice receiving BI2536 monotherapy generally exhibited higher levels of spontaneous activity during routine observation.

Survival analysis demonstrated differences among treatment groups in the intrapulmonary model (Figure 5c). Pairwise comparison using the Log-rank (Mantel–Cox) test revealed a significant increase in survival of the Tumor + BI2536 group when compared to the Tumor + vehicle group (*p* < 0.05). Additionally, the Tumor + BI2536 group also showed a significant improvement in survival compared with the Tumor + Navitoclax group (*p* < 0.01). No statistically significant differences in survival were observed among the remaining group comparisons. Although mortality occurred in all experimental groups, the Tumor + BI2536 group showed significantly longer survival than the Tumor + Vehicle and Tumor + Navitoclax groups. Macroscopic evaluation of lung tumors revealed rapid and aggressive tumor progression in the intrapulmonary model (Figure 5d). Representative images show the presence of small tumor nodules at early stages, followed by rapid expansion into large tumor masses occupying extensive areas of the lung parenchyma at later time points. For further macroscopic quantification, animals were categorized as without tumor, small/moderate tumor, or large tumor (Figure 5e). Contingency analysis revealed a significant association between treatment group and tumor burden category (Fisher–Freeman–Halton test, *p* = 0.02521). Macroscopic tumor burden was assessed at necropsy in animals that died before the scheduled endpoint as well as in animals euthanized at the scheduled endpoint. Pairwise comparisons demonstrated that both BI2536 and Navitoclax monotherapy differed significantly from vehicle-treated animals (*p* < 0.05), whereas the combination therapy did not reach statistical significance compared with vehicle. In fact, the Tumor + BI2536 group exhibited the highest number of animals with no visible tumor and the lowest incidence of large tumors, whereas all animals in the Tumor + Vehicle group presented large tumors. Representative photographs of the presence or absence of macroscopic tumor phenotypes (small, moderate and large tumors) are shown in Figure 5f.

Histopathological evaluation of lung tissues (Figure 5g) confirmed aggressive tumor characteristics across all experimental groups. Neoplastic cells displayed marked cellular and nuclear pleomorphism, increased nucleocytoplasmic ratio, prominent nucleoli, and numerous mitotic figures, consistent with high proliferative activity. Pulmonary histopathology revealed solid growth patterns with high cellularity in the inoculated groups. Lesions exhibited partial replacement of normal lung parenchyma and compression of adjacent alveolar spaces. No apparent morphological differences were observed between the treatment groups and the vehicle group in terms of architectural pattern, cellularity, or cytological features of the neoplasm.

Regarding systemic toxicity, histopathological evaluation of major organs revealed no evident treatment-related alterations. Heart, kidney, and brain tissues exhibited preserved morphology without signs of inflammation, degeneration, or other pathological abnormalities across the experimental groups. In contrast, hepatic analysis revealed the presence of diffuse hepatic steatosis in tumor-bearing animals. Although no statistically significant differences were detected among groups, a higher frequency of diffuse hepatic steatosis was observed in the Tumor + Nav/BI2536 group, whereas the Tumor + BI2536 group presented the lowest proportion of this alteration (Figure 5i).

Together, these results demonstrate that the intrapulmonary LLC1 model represents a highly aggressive form of lung cancer, characterized by rapid tumor growth, systemic deterioration, and high mortality. Treatment with BI2536 significantly affected body weight dynamics, reducing the extent of weight loss observed during disease progression, and was associated with improved survival.

## 3. Discussion

In the present study, we evaluated the antitumor efficacy and systemic impact of PLK-1 inhibition (BI2536), alone or in combination with the BCL-2 family inhibitor Navitoclax, across complementary murine models of lung cancer. These models capture distinct aspects of tumor growth and clinical relevance, ranging from the easier-to-measure subcutaneous setting to the more clinically representative orthotopic intrapulmonary environment. Our findings reveal model-dependent differences in therapeutic response and highlight both the promise and limitations of targeting PLK-1 and apoptotic pathways in lung cancer.

Cell fate during prolonged mitotic arrest is determined by the interplay between the duration of spindle assembly checkpoint (SAC) activation, BCL-xL activity, and cyclin B1 degradation. Sustained SAC signaling increases the likelihood of apoptosis, whereas BCL-xL serves as a key regulator of the apoptotic threshold. Reduced BCL-xL activity, either through low expression or pharmacological inhibition, facilitates apoptosis during mitosis. In contrast, elevated BCL-xL levels enable cells to survive long enough for cyclin B1 to decline below the threshold required for mitotic exit, resulting in mitotic slippage [24]. Consequently, the addition of Navitoclax is expected to inhibit BCL-xL, lower the apoptotic threshold, and promote cell death during mitotic arrest. This strategy aimed to give a new opportunity to antimitotic drugs that were originally developed to reduce the systemic toxicity associated with antimicrotubule agents by selectively targeting proteins essential for mitosis, but which ultimately demonstrated limited clinical efficacy as monotherapy [20]. Mechanistically, our previous study demonstrated that combining PLK-1 inhibition with apoptotic potentiators, such as Navitoclax, in human lung cancer cells, produces strong synergistic effects in vitro, including in three-dimensional spheroid models that more accurately recapitulate the tumor microenvironment. In these systems, Navitoclax markedly reduced mitotic slippage, a known form of cancer resistance to drugs that induce mitotic arrest, and shifted cell fate toward death during mitosis at lower doses of both agents, providing a clear rationale for combination therapy [23]. Consistent with these findings, the present study further demonstrated that the BI2536/Navitoclax combination also exerted synergistic effects in murine LLC1 lung cancer cells. Moreover, the combination exhibited greater cytotoxicity toward LLC1 cells than toward HEK-293 cells under the experimental conditions tested. However, despite these encouraging preclinical data, our results highlight the challenges of translating in vitro synergy to in vivo efficacy. However, despite these encouraging preclinical data, our results highlight the challenges of translating in vitro synergy to in vivo efficacy.

In the subcutaneous model, BI2536 and Navitoclax, whether alone or in combination, significantly inhibited tumor growth compared to vehicle-treated controls, consistent with the antimitotic effects observed in our in vitro experiments. In a previous study, intraperitoneal administration of the PLK-1 inhibitor MCC1019 at 20 or 40 mg/kg inhibited tumor growth by 51.1% and 68.1%, respectively, after 16 days of treatment in LLC-1-bearing mice [25]. Although both MCC1019 and BI2536 are PLK-1 inhibitors, we observed a tumor volume reduction with BI2536 of 57.36% after administration for only 5 consecutive days at a dose of 10 mg/kg, highlighting the antitumor efficiency of this compound under this specific schedule. However, in our study, the combination of BI2536 with Navitoclax did not significantly outperform monotherapy at the experimental endpoint. Although Navitoclax was expected to potentiate BI2536-induced cell death, the addition of Navitoclax did not result in a higher frequency of central necrosis or apoptotic figures compared with BI2536 monotherapy at the experimental endpoint. One possible explanation is that histopathological evaluation was performed only at the end of the experimental protocol, providing a single morphological snapshot of the tumors. As Navitoclax lowers the apoptotic threshold through inhibition of BCL-xL, it has been shown to accelerate apoptosis during prolonged mitotic arrest rather than simply increase the number of apoptotic cells observed at a given time point [26]. Consequently, apoptotic cells may have already undergone clearance before tissue collection, rendering apoptotic figures less evident in routine histopathological sections collected at the experimental endpoint.

In the more clinically relevant intrapulmonary model, BI2536 treatment was associated with attenuated body weight loss and improved survival during the experimental period, but the combination did not enhance survival beyond BI2536 monotherapy. A previous study showed that the combination of the BCL-2 family inhibitor (Venetoclax) with volasertib (PLK-1 inhibitor) improved survival in leukemia animal models; however, the total drugs administrations were 20 intraperitoneal injections, 4-fold higher than in our study [27]. Interestingly, although the Tumor + Navitoclax group showed smaller macroscopic tumors compared with the Tumor + Vehicle group, these animals did not exhibit reduced survival. This apparent discrepancy likely reflects early mortality in the Navitoclax-treated animals, which died before tumors could reach the larger sizes observed in other groups. Consequently, a higher proportion of Navitoclax-treated animals were classified as having small or moderate tumors at the time of organ collection, despite the presence of aggressive disease. Even though no overt systemic toxicities were observed in mice receiving Navitoclax, animals treated with Navitoclax-containing regimens appeared more apathetic and exhibited reduced spontaneous movement. Therefore, we cannot exclude that Navitoclax-associated effects contributed to early mortality. However, since no significant difference in survival between the different treatments was observed for the other models in the study, it is possible that the cause for the higher mortality in the Navitoclax-treated group might arise from a combination of the effects of the drug and the inherent characteristics of the model. Nevertheless, the attenuation of body weight loss observed in BI2536-treated animals should be interpreted in conjunction with the survival data, as differential mortality among treatment groups may have influenced the number of animals available for body weight assessment at later time points. The intrinsic characteristics of the orthotopic intrapulmonary model should also be considered when interpreting these findings. In our study, tumors displayed rapid expansion and locally invasive growth, extending beyond the pulmonary parenchyma into adjacent structures such as the mediastinum. This extensive local invasion likely contributed to the pronounced clinical deterioration and increased mortality observed, underscoring the orthotopic intrapulmonary model as a particularly aggressive and clinically relevant platform for therapeutic evaluation.

Although BI2536 and the combination therapy did not significantly improve survival or histopathological tumor burden in the intranasal model, immunohistochemical analysis demonstrated a reduction in Ki-67-positive tumor cells in both treatment groups compared with vehicle-treated animals. This finding suggests that PLK1 inhibition was associated with reduced tumor cell proliferative activity even in the absence of measurable improvements in the evaluated phenotypic endpoints. Similar reductions in Ki-67 expression following PLK1 inhibition have been reported in previous preclinical studies, supporting Ki-67 as a pharmacodynamic marker of the antiproliferative effects of PLK1-targeted therapies [28,29]. Collectively, these findings indicate that inhibition of tumor cell proliferation may precede detectable changes in tumor burden or survival, particularly in aggressive orthotopic settings where multiple biological factors influence treatment response.

The differential therapeutic responses observed across the three experimental models likely reflect inherent biological differences between ectopic and orthotopic tumor models rather than intrinsic resistance to PLK1 inhibition. Compared with subcutaneous tumors, orthotopic lung tumors develop within the native pulmonary microenvironment, where tumor–stroma interactions, extracellular matrix composition, immune cell populations, vascularization, and local physiological conditions are better preserved and are known to influence therapeutic responses. Likewise, intranasal inoculation results in multifocal tumor establishment throughout the lung, increasing spatial tumor heterogeneity and potentially contributing to variable therapeutic responses. Although these factors were not directly investigated in the present study, they provide biologically plausible explanations for the model-dependent efficacy observed and further emphasize the importance of model selection in preclinical therapeutic evaluation [30,31]. Consistent with these biological differences, the orthotopic intrapulmonary model was associated with substantially higher mortality than the subcutaneous and intranasal models, which exhibited minimal mortality throughout the experimental period. This observation highlights the particularly aggressive nature of the orthotopic model. Importantly, the limited impact on survival observed in the subcutaneous and intranasal models should not be interpreted as failure of tumor establishment or absence of pulmonary disease. Histopathological analyses confirmed the presence of tumor tissue in the lungs of animals from both models, demonstrating successful tumor engraftment. Rather, compared with the orthotopic intrapulmonary model, tumor progression in these models remained predominantly confined to the pulmonary parenchyma and was associated with less severe clinical deterioration during the experimental period. In the subcutaneous model, the combination treatment uniquely induced systemic hepatic effects, including steatosis, which may reflect drug-induced alterations in lipid metabolism or hepatotoxicity secondary to the combined targeting of PLK-1 and BCL-2 pathways. Drug-induced liver injury, including fatty liver changes and steatosis, has been described as a histopathological manifestation of toxicity associated with diverse pharmacological agents [32]. For instance, intravenous administration of BI2536 has been associated with reversible elevations in liver enzyme levels in patients with advanced solid tumors [33]. On the contrary, a single 50 mg dose of Navitoclax did not increase hepatotoxicity in patients with myelofibrosis and mild or moderate hepatic impairment. Moreover, Navitoclax has been reported to reduce liver fibrosis by selectively inducing apoptosis in senescent hepatic cells, and treatment at 100 mg/kg/day in an oral cancer xenograft model produced no apparent hepatic or renal toxicity [34,35,36]. However, its combination with gemcitabine in patients with solid tumors was associated with elevated alanine aminotransferase (ALT) and aspartate aminotransferase (AST) levels, including grade 3 AST elevations in two patients [37]. Elevated liver enzymes, particularly ALT, are well-recognized indicators of hepatocellular injury and are commonly associated with non-alcoholic fatty liver disease [38]. As both BI2536 and Navitoclax undergo hepatic metabolism, it is possible that their combined administration may increase the risk of hepatic injury [33,35]. Additionally, the components of the vehicle (e.g., DMSO, PEG-400, and Tween-80) have been associated with hepatic alterations in rodents under certain conditions, which may contribute to the observed effects, particularly in the intrapulmonary model [39,40]. However, studies using a vehicle formulation containing these three components for in vivo drug delivery did not report any vehicle-associated toxicity, including hepatotoxicity. For instance, a study using a vehicle consisting of 10% DMSO, 45% stroke-physiological saline solution, 40% PEG400 and 5% Tween-80 in a mouse pneumonia model did not report any evidence of hepatic toxicity. Moreover, 0.1% PEG400/Tween-80 in saline containing an equivalent amount of DMSO evaluated in an HCT116 xenograft model did not cause significant alterations in serum total bilirubin, a marker of liver and biliary function, or in AST and ALT. In fact, all these proteins remained within the normal reference levels [41]. Furthermore, no toxicity was reported for vehicle formulations containing DMSO, PEG400, and Tween-80 at concentrations ranging from 2.5 to 10%, 5–30%, and 0.5–5%, respectively [42,43,44]. Nonetheless, in the present study, in addition to the DMSO-containing vehicle, a hydrochloric acid-based vehicle was used for BI2536 administration. Thus, we cannot exclude the possibility that the combined use of both vehicles may have contributed to the observed hepatotoxicity. Importantly, in the present study, the Tumor + Vehicle group received the combined vehicle formulations corresponding to both BI2536 and Navitoclax following the same administration schedule as the treatment groups, and no hepatic steatosis was observed in these animals. This finding is consistent with the studies described above and further supports the interpretation that the vehicle formulation itself is unlikely to be the primary cause of the hepatic alterations observed following combination therapy. Nevertheless, the interpretation of these findings should be made with caution. Moreover, tumor-bearing mice can develop cancer-associated cachexia, with increased tumor burden contributing to the severity of this condition. Cachexia can, in turn, promote metabolic alterations, including increased lipid accumulation, which may contribute to hepatic steatosis [45,46,47]. In this sense, it is possible that tumor burden and the associated cachectic state may have contributed to the hepatic steatosis observed in this study. However, as non-tumor-bearing animals treated with BI2536 and/or Navitoclax were not included in the subcutaneous and intrapulmonary models, the relative contribution of treatment-related toxicity and tumor-associated systemic alterations cannot be fully distinguished. Therefore, although the absence of hepatic steatosis in the Tumor + Vehicle group argues against a major contribution of the vehicle formulation, the hepatic alterations observed following combination therapy may reflect treatment effects, tumor-associated metabolic changes, or an interaction between both factors. Future studies incorporating healthy treated controls will be required to better define the hepatic safety profile of this therapeutic combination.

All these findings suggest that in vivo factors such as differential drug distribution, tumor microenvironment influences, immune responses, and systemic toxicity may attenuate potential synergistic effects observed in vitro. For instance, the synergistic interaction between BI2536 and Navitoclax may depend on maintaining a specific drug concentration ratio. While this ratio is easier to control in vitro, the in vivo translatability of drug combinations is often complicated by differences in the pharmacokinetic and pharmacodynamic properties of the individual agents. Variations in drug absorption, distribution, metabolism, and clearance alter the relative concentrations of each drug that reach the tumor, resulting in dynamic changes in their intratumoral ratio [48,49]. Consequently, tumor cells may be exposed to suboptimal BI2536/Navitoclax ratios that are insufficient to reproduce the synergistic apoptotic response observed in vitro, despite each drug retaining individual biological activity. The tumor microenvironment may also contribute to the limited in vivo efficacy of the combination through adaptive responses within the tumor microenvironment, including immune-mediated survival signals and compensatory activation of pro-survival pathways, which may attenuate the apoptotic effects of BI2536 and Navitoclax despite the synergistic activity observed in cultured tumor cells [50]. For instance, sensitivity to Navitoclax has also been associated with the expression level of the anti-apoptotic protein MCL-1. Specifically, higher MCL-1 expression has been correlated with decreased sensitivity to Navitoclax [51]. Therefore, multiple biological processes present in vivo, but absent or poorly represented in conventional in vitro models, might influence the combinatorial effects.

Our data align with broader evidence that combination strategies involving PLK-1 inhibitors may require careful optimization of dosing and scheduling, and potentially incorporation with other modalities such as targeted inhibitors (e.g., PI3K/mTOR inhibitors) which have shown synergistic effects in preclinical NSCLC models [52]. Furthermore, the aggressiveness of orthotopic intrapulmonary tumors in our study underscores the importance of model selection in preclinical evaluation. Orthotopic lung tumors more faithfully mimic the clinical and systemic impact of human disease, characterized by rapid progression, weight loss, and poor survival, compared to ectopic subcutaneous tumors and intranasal tumor inoculation. This aligns with previous reports that tumor microenvironment, organ-specific interactions, and immune factors significantly influence therapeutic outcomes [53].

Nonetheless, this study has several limitations. First, although immunohistochemical analysis of Ki-67 was performed in the intranasal lung cancer model and demonstrated reduced tumor cell proliferation following treatment with BI2536 alone or in combination with Navitoclax, additional pharmacodynamic biomarkers, including cleaved caspase-3 and phospho-CDC25C or phospho-histone H3, were not evaluated. These biomarkers would have provided direct evidence of target engagement by BI2536 and Navitoclax by assessing proliferation, apoptosis, and PLK1 inhibition/mitotic arrest, respectively. Despite this, our conclusions are supported by robust phenotypic endpoints, including tumor growth inhibition, survival outcomes, and histopathological findings, such as necrosis and apoptotic morphology, which are well-established indicators of antitumor activity in vivo. Furthermore, previous preclinical studies evaluating PLK1 inhibitors and Navitoclax have successfully used Ki-67, cleaved caspase-3, and phospho-histone H3 as pharmacodynamic biomarkers in tumor tissues, supporting their relevance in this setting [54,55,56,57]. Another limitation is the absence of plasma and intratumoral drug concentration measurements to confirm drug exposure. Nevertheless, previous preclinical studies have demonstrated that intraperitoneal administration of BI2536 at 10 mg/kg produces significant antitumor activity in murine models and induces pharmacodynamic effects consistent with PLK1 inhibition, including mitotic arrest and increased phospho-Histone H3 staining [55,58,59]. Similarly, Navitoclax (ABT-263) at 50 mg/kg has been widely employed in preclinical murine studies across different experimental models and administration routes, including intraperitoneal injection and oral gavage. At this dose, Navitoclax has consistently demonstrated biological activity compatible with effective inhibition of BCL-2 family proteins, resulting in modulation of apoptotic responses and antitumor effects in vivo. Moreover, pharmacokinetic studies have shown that administration of Navitoclax at 50 mg/kg produces systemic drug exposure and tissue distribution sufficient to elicit its expected biological effects [60,61,62,63]. Both agents have also been shown to exhibit adequate systemic bioavailability and tissue distribution following intraperitoneal administration, including distribution to the lungs, supporting their use in pulmonary tumor models. Although drug concentrations were not directly measured in the present study, the marked antitumor effects observed strongly suggest that therapeutically effective drug exposure was achieved. In addition, hematological parameters, particularly platelet counts, were not evaluated despite thrombocytopenia being a well-recognized adverse effect of Navitoclax. Nevertheless, no overt clinical signs of severe hematological toxicity, such as bleeding, marked lethargy unrelated to tumor burden, or acute distress, were observed during daily monitoring. Another limitation is that Navitoclax monotherapy was not included in the intranasal lung cancer model. Although Navitoclax was evaluated as a single agent in both the subcutaneous and intrapulmonary models, where it exhibited limited antitumor efficacy, the absence of a corresponding monotherapy group in the intranasal model precludes a direct within-model comparison between Navitoclax alone and the combination treatment. Consequently, the specific contribution of Navitoclax monotherapy in this model cannot be fully determined and should be addressed in future studies.

Importantly, the relatively small number of animals included, particularly in the subcutaneous and intranasal models (*n* = 4–5 per group), may have reduced the statistical power to detect moderate treatment effects. Consequently, the absence of statistically significant differences in some comparisons should not necessarily be interpreted as evidence of lack of biological activity. Future studies including larger cohorts will be important to validate these findings and more robustly define the therapeutic efficacy of the combination across different NSCLC models.

Future studies should also provide assessment of immune infiltration markers such as CD8+ T cells and PD-L1 expression to evaluate possible treatment-induced changes in the tumor immune microenvironment. While immune profiling was not assessed in our models, previous studies have suggested that PLK1 inhibition may indirectly influence tumor immune signaling pathways, including modulation of inflammatory and stress-response signaling, which can impact immune cell recruitment and tumor immunogenicity [17]. Additionally, optimization of treatment schedules and extension of the experimental observation period may provide a more comprehensive understanding of the long-term therapeutic effects of BI2536 and its combination with Navitoclax. Such approaches may also help determine whether prolonged drug exposure or alternative dosing regimens improve the translation of the synergistic effects observed in vitro into meaningful antitumor efficacy in vivo.

Taken together, our findings substantiate that PLK-1 inhibition has antitumor potential and can modulate systemic disease progression, particularly in aggressive orthotopic settings. However, the translation of in vitro synergy with apoptotic potentiators such as Navitoclax to in vivo benefit remains complex and may require additional therapeutic partners, optimized regimens, or biomarkers predictive of response.

## 4. Materials and Methods

### 4.1. Reagents and Cell Culture

BI2536 (PLK-1 inhibitor) and Navitoclax (ABT-263) were purchased from MedChem Express (Shanghai, China) and reconstituted in sterile dimethyl sulfoxide (DMSO; Sigma-Aldrich Co., Ltd., St. Louis, MO, USA) to obtain stock solutions at a concentration of 700 mg/mL of Navitoclax and 100 mg/mL of BI2536. To preserve physicochemical stability and biological activity, stock solutions were aliquoted and stored at −20 °C. On the day of use, compounds were freshly diluted in the appropriate vehicle for in vivo administration.

The murine Lewis lung carcinoma cell line (LLC1) was obtained from the Banco de Células do Rio de Janeiro (BCRJ, Rio de Janeiro, Brazil). LLC1 cells were cultured in RPMI-1640 medium supplemented with 2 mM L-glutamine, 10% fetal bovine serum (GIBCO BRL, Grand Island, NY, USA), 100 U/mL penicillin, and 100 μg/mL streptomycin (GIBCO BRL, Grand Island, NY, USA). Human embryonic kidney cells (HEK-293) were kindly provided by Prof. Marcel Leist (University of Konstanz, Konstanz, Germany) and maintained in high-glucose Dulbecco’s Modified Eagle Medium (DMEM) supplemented with 10% fetal bovine serum (GIBCO BRL, Grand Island, NY, USA), 100 U/mL penicillin, and 100 μg/mL streptomycin (GIBCO BRL, Grand Island, NY, USA). All cell cultures were maintained at 37 °C in a humidified incubator containing 5% CO_2_ and were routinely tested to confirm the absence of mycoplasma contamination.

### 4.2. MTT Cell Viability Assay

To evaluate the cytotoxic effects of Navitoclax and BI2536, alone or in combination, HEK-293 and LLC1 cells were seeded at a density of 10,000 cells per well in 96-well plates containing complete culture medium and allowed to adhere overnight at 37 °C in a humidified atmosphere of 5% CO_2_. After 24 h, Navitoclax (31.25, 62.5, 125, 250, and 500 μM) and BI2536 (0.0001, 0.001, 0.01, 0.1, and 9.85 μM), either alone or in combination, were added to the cultures at the indicated concentrations. Following 48 h of treatment, cell viability was determined using the 3-(4,5-dimethylthiazol-2-yl)-2,5-diphenyltetrazolium bromide (MTT) assay. Briefly, MTT solution was added to each well to achieve a final concentration of 0.25 mg/mL, followed by incubation for 4 h at 37 °C. The resulting formazan crystals were dissolved in acidified isopropanol (0.04 M HCl), and absorbance was measured at 570 nm using a Varioskan™ LUX multimode microplate reader (Thermo Fisher Scientific, Waltham, MA, USA).

All experiments were performed in two independent biological experiments, each conducted with three technical replicates, and cell viability was expressed as a percentage relative to untreated control cells. Dose–response curves and half-maximal inhibitory concentration (IC_50_) values were determined by nonlinear regression using GraphPad Prism version 8 (GraphPad Software Inc., San Diego, CA, USA).

### 4.3. Animals and Ethical Approval

Male C57BL/6J mice were obtained from the UFMG central animal facility and also kindly donated by Instituto René Rachou/Fiocruz (Belo Horizonte, Brazil). Animals were housed under specific pathogen-free conditions at the Department of Physiology and Biophysics under controlled environmental conditions (12 h light/dark cycle, temperature 22 ± 1 °C) with ad libitum access to food and water. Upon arrival from the central animal facility, animals were randomly distributed among cages and maintained under these conditions throughout the acclimatization period. All tumor inoculations were performed when animals reached 11–12 weeks of age. Before treatment initiation, the cages were randomly assigned to the experimental treatment groups, such that all animals within the same cage received the same treatment. The exact sample size (n) for each experimental group and experimental model is reported in the corresponding figure legends. Sample sizes were defined a priori for each experimental model in accordance with the ethical principle of Reduction, aiming to minimize animal use while ensuring adequate biological assessment. No predefined inclusion or exclusion criteria other than those specified in the experimental protocol were applied. No animals, experimental units or data points were excluded from the analyses. All animals allocated to each experimental group were included in the corresponding statistical analyses. Differences in group size between experiments reflect independent experimental cohorts designed for distinct experimental models and were not the result of post hoc exclusion of animals. To minimize potential confounding factors, all animals were maintained under identical environmental conditions and handled according to the same experimental protocol. All treatments were administered during the morning, and the position of the cages within the animal room was periodically alternated throughout the study to minimize potential location-related effects…

The outcome measures assessed in this study included body weight, tumor burden, overall survival, histopathological findings (including tumor necrosis/apoptosis and hepatic steatosis) and Ki-67 immunostaining, according to the experimental model. All experimental procedures were conducted in accordance with the National Institutes of Health (NIH) guidelines for the care and use of laboratory animals and were previously approved by the Ethics Committee on Animal Use of the Federal University of Minas Gerais (CEUA-UFMG; protocol number 185/2024). All efforts were made to minimize animal suffering and distress throughout the study. Each animal was considered to be an experimental unit.

### 4.4. Mouse Models of Lung Cancer

Sub-confluent LLC1 cultures were harvested using 0.05% trypsin–EDTA (Thermo Fisher Scientific Inc., Waltham, MA, USA), washed with phosphate-buffered saline (PBS), and resuspended as single-cell suspensions at a concentration of 1 × 10^6^ cells per 100 μL PBS.

For subcutaneous tumor induction, on day 0, mice were first anesthetized via intraperitoneal injection of ketamine (100 mg/kg; Cristália, São Paulo, Brazil) and xylazine (5 mg/kg; Rompun^®^, Bayer, Leverkusen, Germany). After anesthesia, the left flank region was shaved and aseptically prepared, and approximately 1 × 10^6^ LLC1 cells suspended in 100 μL of PBS were injected subcutaneously into the left flank of the mice to facilitate tumor monitoring. Tumor growth was assessed using digital calipers, and tumor volume was calculated based on the following formula: *V* = (*L* × *W*^2^)/2 where *V* is the volume (mm^3^), *L* is the largest diameter (mm), and W is the smallest diameter (mm). Longitudinal assessment of tumor volume was performed exclusively in the subcutaneous model, where tumor growth could be monitored externally throughout the experimental period. In the intranasal and intrapulmonary models, tumor growth occurred within the lung parenchyma and therefore could not be monitored serially during the experimental period. For intranasal tumor induction, mice were anesthetized with ketamine (100 mg/kg) and xylazine (5 mg/kg). Subsequently, 1 × 10^6^ LLC1 cells suspended in 100 μL PBS were slowly administered intranasally.

For intrapulmonary tumor induction, mice were anesthetized via intraperitoneal injection of ketamine (100 mg/kg) and xylazine (5 mg/kg). An incision (1 cm) was made on the left side of the chest, and soft tissues were gently dissected to expose the thoracic ribs and intercostal space, as previous described [64]. LLC1 cells (1 × 10^6^ cells suspended in 100 μL PBS) were injected directly into the left lung parenchyma using a 29-gauge syringe needle. The incision site was sutured, and animals were allowed to recover under a warming lamp until fully awake. Macroscopic evaluation of pulmonary tumor burden was performed only in the intrapulmonary model, in which tumors were readily visible at the experimental endpoint. In contrast, tumors induced by the intranasal route were not macroscopically detectable and could only be identified by histopathological examination.

### 4.5. Drug Treatment Protocols

Following tumor establishment, animals were randomly allocated to receive vehicle alone, Navitoclax (50 mg/kg), BI2536 (10 mg/kg), or the combination of Navitoclax (50 mg/kg) and BI2536 (10 mg/kg), using a simple randomization procedure. All treatments were administered via intraperitoneal injection once daily for five consecutive days.

In the subcutaneous tumor model, treatment was initiated when tumors reached an average volume of approximately 150–200 mm^3^, which occurred on day 12 after cell inoculation. Treatment continued until day 16, and animals were euthanized on day 17.

In the intrapulmonary tumor model, LLC1 cells were inoculated on day 0, and treatment was initiated on day 7, continuing for five consecutive days until day 11. Animals were euthanized on day 12.

In the intranasal tumor model, LLC1 cells were inoculated on day 0, and treatment was initiated on day 25, following the same five-day treatment regimen.

Navitoclax was formulated in a vehicle containing 10% DMSO, 40% PEG-400, 10% Tween and 40% saline, while BI2536 was prepared in a hydrochloric acid-based vehicle (0.1 HCl, 0.9% NaCl). Vehicle-treated animals received the corresponding vehicles for both Navitoclax and BI2536, following the same administration schedule as the treatment groups. At the time of euthanasia, lungs, brain, spinal cord, spleen, kidneys, liver, heart, and primary tumors (subcutaneous model) were collected for subsequent histopathological analyses.

During the experimental period, animals were monitored daily for body weight, survival, tumor progression, and general clinical condition. Body weight was recorded at predefined time points and expressed as relative variation from baseline (day 0). Clinical evaluation included cage activity (locomotion and interaction with the environment), as well as signs of distress or impaired welfare, such as piloerection, apathy, reduced mobility, respiratory compromise, inability to access food or water, excessive tumor burden, tumor ulceration, or any other condition considered incompatible with animal welfare. Whenever animals exhibited signs suggestive of reduced mobility or decreased activity, food was placed directly on the cage floor to facilitate access and ensure adequate food intake, thereby minimizing unnecessary distress. Humane endpoint criteria were predefined in accordance with the approved institutional animal ethics protocol and included signs of severe distress or any condition considered incompatible with animal welfare. Animals meeting these criteria were scheduled for humane euthanasia before the planned experimental endpoint. However, no animals reached the predefined humane endpoint criteria during the study. Animals either died spontaneously during disease progression or were euthanized at the planned experimental endpoint according to the respective experimental protocol.

### 4.6. Histological Analysis

At the defined experimental endpoints, mice were anesthetized with ketamine (100 mg/kg) and xylazine (5 mg/kg) and subjected to transcardiac perfusion with 10 mL saline, followed by 10 mL of 10% buffered formalin (pH 7.2). Samples of primary tumor and organs were collected (trachea, heart, lung, liver, kidney and brain), fixed in buffered formalin, routinely processed, embedded in paraffin, and sectioned at a thickness of 4 μm.

Histological sections were dewaxed in xylene, rehydrated through graded ethanol solutions, and stained with hematoxylin for 5 min, and counterstaining with eosin was performed for 30 s. Sections of primary tumor and organs were dehydrated, cleared and mounted with Entellan^®^. All histopathological evaluations, including the assessment of tumor necrosis, apoptosis, hepatic steatosis, and Ki-67 immunostaining, were performed independently by two trained pathologists blinded to treatment allocation. Images were acquired using a digital camera coupled to the optical microscope (Olympus BX-40; Olympus, Tokyo, Japan).

### 4.7. Immunohistochemical

For the immunohistochemical reaction, 4 μm thick histological sections were obtained from lungs containing primary tumors and mounted on slides for subsequent analysis. Antigen detection was performed using the Novolink Polymer Detection System (Leica Biosystems, Newcastle Upon Tyne, UK), following the manufacturer’s recommendations. Endogenous peroxidase activity was blocked with a 10% hydrogen peroxide (H_2_O_2_) solution in methanol for 30 min. To block non-specific binding, slides were incubated with a 1% bovine serum albumin (BSA) solution combined with a 3% milk solution for 30 min, followed by incubation with a 2% BSA solution for an additional 30 min. Reagents were applied manually, and immunoreactivity was visualized by incubation with the 3,3′-diaminobenzidine (DAB) chromogen (DAB Substrate System, Dako, Carpinteria, CA, USA) for 1 min. Primary antibody specifications, including dilutions, antigen retrieval methods, and incubation times, are described in Table 3. Photomicrograph acquired at 40× magnification from the hotspot (area with the highest staining density). A total of 500 cells per sample were counted, and marker expression was expressed as the percentage of positive cells. Quantification was performed by light microscopy analysis in representative hotspot areas, and the percentage of positive cells was calculated as the number of immunoreactive cells divided by the total number of counted cells.

### 4.8. Statistical Analysis

Data are presented as mean ± standard error of the mean (SEM). Statistical analyses were performed using GraphPad Prism software (version 8.0; GraphPad Software, San Diego, CA, USA) and R software (version 4.5.2; R Foundation for Statistical Computing, Vienna, Austria). Normality of data distribution was assessed using the Shapiro–Wilk test before performing parametric analyses. For continuous datasets involving comparisons among more than two groups, one-way or two-way analysis of variance (ANOVA) was applied as appropriate, followed by Tukey’s multiple-comparisons post hoc test. Repeated measurements of tumor growth and body weight were analyzed using mixed-effects models. Mixed-effects models were used for longitudinal analyses of tumor growth and body weight because they account for within-animal correlations and allow the inclusion of incomplete observations resulting from biological variability and predefined humane endpoints. Ki-67 immunohistochemical quantification was analyzed using one-way ANOVA followed by Tukey’s multiple-comparisons test. Survival curves were generated using the Kaplan–Meier method and compared using the Log-rank (Mantel–Cox) test. Pairwise survival comparisons were performed between groups as appropriate and were considered exploratory; no FDR correction was applied. Macroscopic tumor burden was analyzed using the Fisher–Freeman–Halton test in R, with pairwise comparisons conducted as appropriate. Differences in the frequency of histopathological findings (apoptosis/necrosis and hepatic steatosis) among experimental groups were analyzed using Fisher’s exact test with Monte Carlo simulation, followed by pairwise Fisher tests with false discovery rate (FDR) correction. Differences were considered statistically significant when *p* < 0.05.

## 5. Conclusions

In this study, we established and characterized different murine models of lung cancer generated by subcutaneous, intranasal, and intrapulmonary inoculation of LLC1 cells. These complementary models enabled the evaluation of tumor progression, systemic manifestations, and therapeutic responses under distinct biological conditions, highlighting important differences in disease aggressiveness according to the site of tumor establishment.

Our findings demonstrated that the intrapulmonary model represented the most aggressive pattern of disease progression, characterized by marked body weight loss, reduced survival, and extensive tumor burden. In this model, BI2536 partially attenuated disease progression by reducing body weight loss, improving survival, and decreasing tumor size. In the subcutaneous model, BI2536, Navitoclax, and their combination significantly inhibited tumor growth, whereas BI2536 monotherapy was associated with increased necrosis and apoptotic morphology. In addition, the combination treatment uniquely induced hepatic steatosis. In the intranasal model, no significant differences were observed in survival or body weight among treatment groups; however, immunohistochemical analysis demonstrated that BI2536, either alone or in combination with Navitoclax, significantly reduced the proportion of Ki-67-positive tumor cells, indicating reduced tumor cell proliferation despite the absence of measurable effects on overall disease progression.

Although the combination of BI2536 and Navitoclax produced marked synergistic effects in vitro, these findings were not fully translated to the in vivo setting, emphasizing the complexity of antitumor responses within biologically relevant tumor microenvironments. Factors such as drug pharmacokinetics, intratumoral drug distribution, and microenvironment-dependent survival mechanisms are likely to contribute to these differences and should be further investigated.

Overall, this study provides a comprehensive comparison of complementary preclinical lung cancer models and reinforces the importance of model selection for therapeutic evaluation. Future studies incorporating pharmacokinetic analyses, additional pharmacodynamic biomarkers, optimized treatment schedules, and immune microenvironment characterization will be essential to better define the therapeutic potential of PLK1 inhibition, alone or in combination with apoptosis-targeting strategies, in lung cancer.

## Figures and Tables

**Table 1:** BI2536 and Navitoclax IC_50_ in LLC1.

Drugs	IC_50_ (µM)
BI2536	0.0025 ± 0.0005
Navitoclax	43.21 ± 4.59

**Table 2:** BI2536 and Navitoclax IC_50_ in HEK-293.

Drugs	IC_50_ (µM)
BI2536	>9.85
Navitoclax	74.44 ± 1.75

**Table 3:** Antibodies, dilutions, antigen retrieval procedures and incubation times.

Target Antigen	Clone	Dilution	Antigen Retrieval Method	Incubation Time (h)/Temp
Ki-67	Monoclonal Rabbit Human, Mouse, Rat (SP6)	1:50	Pressurized Heat (125 °C/2 min) with citrate buffer pH 6.0	14–16 h/4 °C

## Data Availability

The original contributions presented in this study are included in the article. Further inquiries can be directed to the corresponding authors.

## Abbreviations

The following abbreviations are used in this manuscript:

°C	Celsius
µm	Micrometers
ALK	Anaplastic Lymphoma Kinase
BCL-2	B-cell Lymphoma 2
BCL-XL	B-cell Lymphoma- Extra Large
BCL-W	B-cell Lymphoma W
DMSO	Dimethyl Sulfoxide
EGFR	Epidermal Growth Factor Receptor
FBS	Fetal Bovine Serum
H&E	Hematoxylin and Eosin
kg	Kilogram
LLC1	Lewis Lung Carcinoma
mg	Milligrams
mL	Milliliters
mm	Millimeters
mTOR	Mechanistic Target of Rapamycin
NIH	National Institutes of Health
NSCLC	Non-small Cell Lung Cancer
PBS	Phosphate-buffered Saline
PI3K	Phosphatidylinositol 3-kinase
PLK-1	Polo-Like Kinase 1
